# Metabolic phenotype-microRNA data fusion analysis of the systemic consequences of Roux-en-Y gastric bypass surgery

**DOI:** 10.1038/ijo.2015.33

**Published:** 2015-04-28

**Authors:** Q Wu, J V Li, F Seyfried, C W le Roux, H Ashrafian, T Athanasiou, W Fenske, A Darzi, J K Nicholson, E Holmes, N J Gooderham

**Affiliations:** 1 Division of Computational and Systems Medicine, Department of Surgery and Cancer Imperial College London, South Kensington, London, UK; 2 Center for Digestive and Gut Health, Institute of Global Health Innovation, Imperial College London, London, UK; 3 Department of General and Visceral, Vascular and Pediatric Surgery, University Hospital of Würzburg, Würzburg, Germany; 4 Diabetes Complications Research Centre, Pathology, Conway Institute, School of Medicine and Medical Sciences, University College Dublin, Dublin, Ireland; 5 Investigative Science, Imperial College London, London, UK

## Abstract

**Background/Objectives:**

Bariatric surgery offers sustained marked weight loss and often remission of type 2 diabetes, yet the mechanisms of establishment of these health benefits are not clear.

**Subjects/Methods:**

We mapped the coordinated systemic responses of gut hormones, the circulating miRNAome and the metabolome in a rat model of Roux-en-Y gastric bypass (RYGB) surgery.

**Results:**

The response of circulating microRNAs (miRNAs) to RYGB was striking and selective. Analysis of 14 significantly altered circulating miRNAs within a pathway context was suggestive of modulation of signaling pathways including G protein signaling, neurodegeneration, inflammation, and growth and apoptosis responses. Concomitant alterations in the metabolome indicated increased glucose transport, accelerated glycolysis and inhibited gluconeogenesis in the liver. Of particular significance, we show significantly decreased circulating miRNA-122 levels and a more modest decline in hepatic levels, following surgery. In mechanistic studies, manipulation of miRNA-122 levels in a cell model induced changes in the activity of key enzymes involved in hepatic energy metabolism, glucose transport, glycolysis, tricarboxylic acid cycle, pentose phosphate shunt, fatty-acid oxidation and gluconeogenesis, consistent with the findings of the *in vivo* surgery-mediated responses, indicating the powerful homeostatic activity of the miRNAs.

**Conclusions:**

The close association between energy metabolism, neuronal signaling and gut microbial metabolites derived from the circulating miRNA, plasma, urine and liver metabolite and gut hormone correlations further supports an enhanced gut-brain signaling, which we suggest is hormonally mediated by both traditional gut hormones and miRNAs. This transomic approach to map the crosstalk between the circulating miRNAome and metabolome offers opportunities to understand complex systems biology within a disease and interventional treatment setting.

## Introduction

Obesity and its comorbidities have reached epidemic proportions across the developed and developing world, imposing an unsustainable socioeconomic burden on many societies.^1^ Bariatric surgery is the most effective treatment strategy for morbidly obese patients (body mass index >40 kg m^−^^2^), or those with obesity comorbidities at a lower body mass index, as it can achieve sustained long-term weight loss and place type 2 diabetes in remission within days.^2^ Unlike diet-induced weight loss, Roux-en-Y gastric bypass (RYGB) achieves substantial weight loss with enhanced satiety, decreased hunger and increased energy expenditure while food restriction and malabsorption have not been identified as major players, in addition to reduced food intake.^3^ However, although the anatomical rearrangement leads to enterohormonal changes, altered bile flow^4^ profound changes of the gut microbiota^5, 6^ and downregulation of endocannabinoids,^7^ the mechanism by which RYGB modulates metabolic pathways in an integrated way and the molecules responsible for coordinating these effects remain largely unknown.

MicroRNAs (miRNAs) are non-coding RNAs, 18–25 nucleotides in length, which regulate thousands of genes at the post-transcriptional level;^8^ each miRNA can potentially target multiple mRNAs. This multi-targeting feature of miRNAs defines their unique role in governing multiple metabolic processes simultaneously. With their ability to influence pathway networks and high expression level in cells, miRNAs are believed to confer biological system robustness under homeostatic disturbance.^9^ We hypothesized that following RYGB surgery, which profoundly changes nutrients and bile flow, the individual establishes a new metabolic balance via the manipulation of miRNAs. Therefore, we profiled and integrated the plasma miRNAome and metabolome from Sprague Dawley (SD) rats undergoing RYGB surgery using a Statistical HeterospectroscopY (SHY) method,^10^ aiming to probe the composition of circulating miRNAs, which could behave as master metabolic regulators mediating post RYGB biological effects. Subsequent downstream *in vitro* model was used to probe these miRNA-mediated metabolic pathway alterations post RYGB surgery.

## Materials and methods

### Experiment design and sample collection

The animal experiment was carried out under a UK home office licence (PL 70-6669). Thirteen male SD rats were individually housed and kept under a 12 h/12 h light/dark cycle at room temperature. All rats were acclimatized and fed high-fat diet for 1 week prior to the experiment. Animals were randomly divided into two groups: *RYGB (n*=8) and *SHAM (n*=5). The antibiotic combination amoxicillin/flucoxacillin was administrated pre-surgery to all rats at a dose of 12.5 mg per rat.

Animal body weight (BW) and food intake were measured daily. The gut hormones (for example, peptide YY (PYY) and glucagon-like peptide 1 (GLP-1)) were measured on the day of killing. Blood was collected from all groups in the fasting state via cardiac puncture under terminal anesthesia in tubes containing EDTA and dipeptidyl peptidase-4 inhibitor. The plasma fraction was separated by centrifugation at 4 °C and stored at −80 ^o^C. All samples were assayed in duplicate. PYY-like inmmunoactivity was measured with a specific and sensitive radioimmunoassay, which measure both the full length (PYY1-36) and the fragment (PYY3-3^). GLP-1 was measured in duplicate by established in-house radioimmunoassay (Millipore, Billerica, MA, USA).

Spot urinary samples were collected at day 52 and all rats were culled 53 days after the surgery. Approximately 5 ml of whole blood was taken from the heart, 2.5 ml of which was transferred into an EDTA-containing tube (BD Bioscience, Oxford, UK) and the remaining to a sodium heparin-coated tube. After a gentle shake, they were immediately centrifuged at 3500 *g* at room temperature for 10 min. The resulting plasma samples were collected into two 1.5-ml RNase-free Eppendorf tubes, separately. The left lobe of the liver was collected from each rat. All samples were immediately snap-frozen in liquid nitrogen and stored at −80 ^o^C.

### Sample preparation for NMR spectroscopic analyses

Plasma samples collected using sodium heparin and urine were thoroughly defrosted and vortexed for 15 s. A total of 30 μl of urine was mixed with 25 μl of 0.2 M sodium phosphate buffer in deuterium oxide (0.01% of sodium 3-(trimethylsilyl) propionate-2,2,3,3-*d*_4_, pH=7.4), and 50 μl of the mixture was transferred into a nuclear magnetic resonance (NMR) tube with an outer diameter of 1.7 mm for further spectroscopic analysis. A total of 400 μl of plasma was mixed with 250 μl of saline containing 20% deuterium oxide for the magnetic field lock. The resulting mixture was centrifuged at 10 000 *g* for 10 min, and 600 μl of supernatant was transferred into a NMR tube with an outer diameter of 5 mm pending ^1^H NMR spectral acquisition. The liver tissue extraction is described in SI Materials and Methods. The dry extracts of liver aqueous phase were resuspended in a mixture of 100 μl water, 400 μl deuterium oxide and 400 μl aforementioned sodium phosphate buffer, centrifuged for 10 min at 10 000 *g*, and 600 μl of supernatant was transferred into a NMR tube.

### ^1^H Nuclear magnetic resonance spectroscopy of urine, plasma and liver extracts, and spectral data analysis

^1^H NMR spectra of urine, plasma and liver aqueous extract samples were obtained using a Bruker 600 MHz spectrometer (Bruker; Rheinstetten, Germany) at the operating ^1^H frequency of 600.13 MHz with a temperature of 300 K. A standard NMR pulse sequence (recycle delay (RD)-90^o^-t_1_-90^o^-t_m_-90^o^-acquisition) was applied to acquire one-dimensional ^1^H NMR spectral data, where *t*_1_ was set to 3 μs and *t*_m_ (mixing time) was set to 100 ms. Suppression of the water peak was achieved using selective irradiation during RD of 2 s and *t*_m_. A 90 degree pulse was adjusted to ~10 μs. A total of 128 scans for plasma samples and 256 scans for liver aqueous extracts were collected into 64 k data points with a spectral width of 20 ppm. A Carr–Purcell–Meiboom–Gill pulse sequence (RD-90°-(τ-180°-τ)_n_-acquisition) was applied additionally to plasma samples to better visualize the signals of the low molecular weight metabolites. For the Carr–Purcell–Meiboom–Gill experiment, a spin relaxation delay (2nτ) of 64 ms was used. ^1^H NMR spectral data processing and multivariate statistical analysis are described in Supplementary Information Materials and Methods.

### RNA isolation, reverse transcription and preamplification for Taqman miRNA low-density array

Total RNAs in plasma and the liver homogenates were extracted using a mirVANA PARIS RNA isolation kit (Ambion, Paisley, UK). The procedures for the liver RNA extraction followed the manufacturer's instruction, whereas for plasma samples, minor modifications were applied to better eliminate plasma protein. In brief, 100 μl of the plasma sample was diluted with 100 μl cell disruption buffer and then 200 μl of 2 × denature solution was added. The remaining extraction steps are the same as the manufacturer's instruction. RNAs from both plasma and liver tissues were eluted from mirVANA PARIS columns using 100 μl of 95 ^o^C DNase and RNase-free water and stored at −20 ^o^C pending analysis.

Total RNA was reverse transcribed using Taqman Megaplex pools. Reverse transcription (RT) mix was prepared following the manufacturer's instructions. RT mix (4.5 μl) was mixed with 3 μl total RNA extracted from plasma. The RT products were preamplified according to the manufacturer's recommendations. RT product (2.5 μl) was combined with 12.5 μl Taqman PreAmp Master Mix (2 × ) and 2.5 μl Megaplex PreAmp Primers (10 × ) to generate a final 25 μl preamplification PCR mix. Preamplification was carried out following the manufacturer's instructions. Preamplification PCR product (10 μl) was diluted with 30 μl 0.1 × TE buffer (pH 8.0). The diluted preamplification PCR product was stored at −20 ^o^C until required.

### Taqman miRNA low-density array

Taqman Array Rodent Card is able to detect all 373 miRNAs expressed in rat according to the Sanger miRBase v15. Each complete assay contain two sets of cards (Card A and B). Card A detects well-characterized miRNAs, which tend to be function defined and broadly/highly expressed. Card B detects most of the recently discovered, less functional defined and narrowly or low-expressed miRNAs. Diluted preamplification product (9 μl) was combined with 450 μl Taqman universal PCR Master Mix (no AmpErase UNG, 2 × ) and 441 μl RNase/DNase-free water. Quantitative PCR reaction mix (100 μl) was loaded into each port of the TaqMan MicroRNA Array. The array card was centrifuged at 331 *g* for 2 min and then mechanically sealed with Applied Biosystems 7900HT upgrade kit. Quantitative RT-PCR reaction was carried out using an Applied Biosystems 7900HT thermocycler according to the manufacturer's recommended cycling conditions.

The single Taqman miRNA assay, mRNA target prediction, protein extraction from the liver and Immunoblot, MiRDIAN miR-122 mimic transfection, and statistical correlation analysis among gut hormones, miRNAome and metabolome are described in SI Materials and Methods.

## Results

### RYGB surgery alters gut hormone levels and metabolite profiles of urine, plasma and liver extracts in SD rats

RYGB surgery induced a reduction of 22.0% of pre-surgical BW in SD rats (*n*=8) over 52 days (pre-surgery BW=467.9±12.9 g, post-surgery BW=377.8±33.9 g), whereas sham-operated animals (*n*=5) had an average BW of 573.2 g over the experimental period (Supplementary Figure S1A). Consistent with previous studies,^11^ we observed that the plasma concentrations of gut hormones, GLP-1 and PYY, were significantly elevated following RYGB surgery (GLP-1_RYGB_=71.5±11.7 pmol l^−1^, GLP-1_SHAM_=10.5±3.3 pmol l^−1^, *P*<0.001; PYY_RYGB_=26.2±5.6 pmol l^−1^, PYY_SHAM_=10.4±4.8 pmol l^−1^, *P*<0.001) (Supplementary Figures S1B and C).

Orthogonal-partial least squares-discriminant analysis (OPLS-DA) of ^1^H nuclear magnetic resonance (NMR) spectroscopic data of urine, plasma and liver tissue collected 52 days post operation showed clear postoperative shift in metabolism in all matrices (Supplementary Figure S2, Table 1). Similar to our previous findings in Wistar rats and C57BL6 mice, urinary concentrations of citrate, fumarate, 2-oxoglutarate and succinate decreased post RYGB surgery, suggesting an enhancement in tricarboxylic acid cycle (TCA) activity.^6, 12^ Increased urinary excretion of host-microbial co-metabolites including indoxyl sulfate, *p*-cresyl sulfate, *p*-cresyl glucuronide, phenylacetylglycine and trimethylamine *N*-oxide were observed. RYGB reduced plasma lipids and increased pyruvate, lactate and alanine, suggesting increased lipid metabolism and glycolysis. Elevations in the concentrations of hepatic glycogen, glucose, glycerol, lactate and alanine are consistent with the downregulation of gluconeogenesis and an increased conversion between glycogen and glucose. These observations of altered hormonal and metabolic status support extant literature and suggest that RYGB extensively regulates multiple energy metabolism-related pathways.

### RYGB profoundly influences the global circulating miRNA expression in plasma

**Figure 1:**
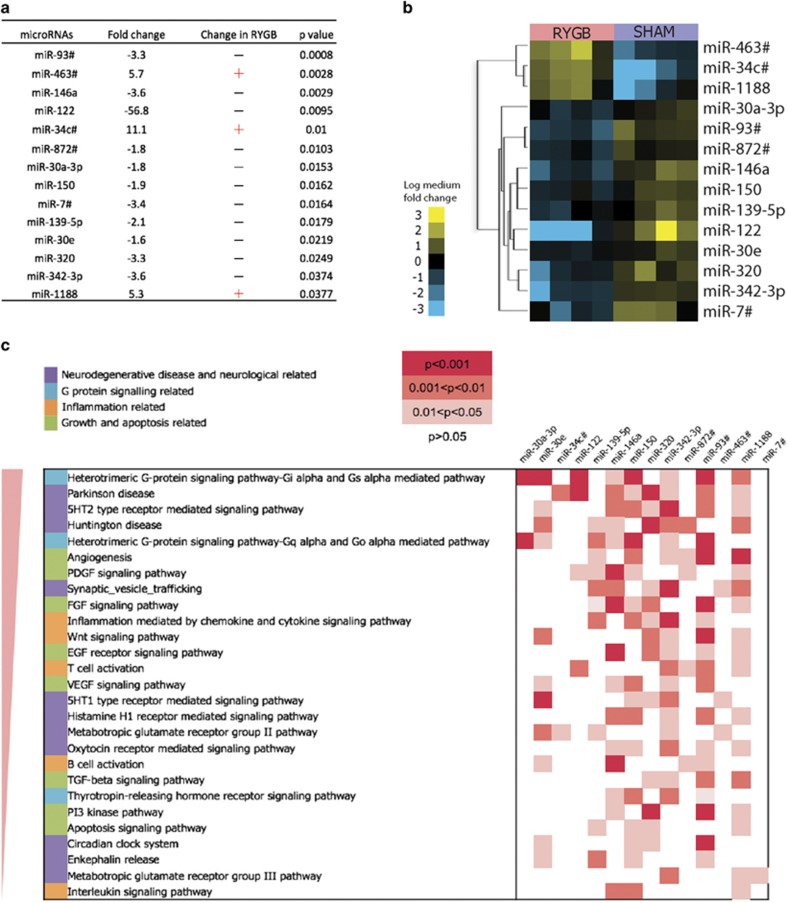
Altered circulating microRNAs and targeted signaling pathways. (**a**) The Roux-en-Y gastric bypass (RYGB) surgery altered the expression of 14 circulating microRNAs with 3 upregulated (yellow) and 11 downregulated (blue) in the RYGB-operated animals (*n*=4) compared with sham controls (*n*=4). *P*-values are derived from two-tailed *t*-test. (**b**) The heat map with two-way clustering shows clear grouping of RYGB and SHAM animals based on 14 significantly altered microRNAs. (**c**) The predicted pathways that these microRNAs can target are ranked according to the number of potentially involved microRNAs. *P*-values represent a statistical overrepresentation or underrepresentation of the genes in the predicted microRNA target list relative to the reference pathway gene list.

We next investigated the influence of RYGB on the circulating miRNAome using Taqman low-density array cards. Normalization is a key issue in circulating miRNA data analysis, thus we first developed a method for normalization of multiple-miRNAs and demonstrated its superiority over the widely used spike-in *Caenorhabditis elegans* cel-miR-39, global normalization and U6 normalization methods (as detailed in the Supplementary information, Supplementary Figure S3). Raw miRNA data were normalized using five endogenous miRNAs (U6-1, U6-2 miR-16, miR-223 and miR-1937b), and a total of 113 and 92 plasma circulating miRNAs were detected in four RYGB- and four SHAM-operated rats, respectively, 88 of which were common to both groups (Supplementary Table S1). Fourteen miRNAs were found to be significantly differentially expressed using a two-tailed student *t*-test (*P*<0.05) (Figure 1a). Eleven of the altered miRNAs were downregulated (miR-122, miR-93*, miR-872, miR-7*, miR-146a, miR-342-3p, miR-150, miR-139, miR-30a, miR-30e, miR-320), whereas three miRNAs, namely miR-463*, miR-34c* and miR-1188, were upregulated in the RYGB group. The clustered miRNA profiles show clear differentiation of the RYGB and SHAM groups (Figure 1b), wherein the RYGB-altered miRNAs typically exhibited a 1.5- to sixfold change compared with sham-operated rats, with the exception of miR-122, which demonstrated a 56-fold (*P*=0.0095) downregulation in RYGB-operated animals. This is consistent with a previous finding in RYGB patients, where miR-122 decreased significantly (−94.2%, *P*<0.0001) when comparing baseline and after surgery miR-122 levels.^13^ Therefore, our data support the hypothesis that RYGB significantly alters circulating miRNAs, and thus the metabolic consequences of this require further investigation.

### RYGB-altered plasma miRNAs influence multiple inflammation, obesity and neurodegenerative disease-related pathways

To gain insight into miRNA-mediated metabolic pathways affected by RYGB surgery, we predicted the mRNA targets of each of these altered circulating miRNAs with nine commonly used databases (See Materials and Methods) and retained only targets predicted by more than two programmes for further analysis. Based on the predicted pathways ranked according to the number of discriminatory miRNAs involved in each pathway (Figure 1c), we found that signaling pathways, specifically G protein signaling, neurodegenerative, inflammation, and growth and apoptosis were highly likely to be influenced by the surgery. In particular, miR-342-3p, miR-320, miR-139-5p and miR-146a were predicted to be involved in multiple neurological transmitter and receptor-related pathways, as well as two major neurodegenerative disease-associated pathways (Parkinson's and Huntingtons diseases), suggesting that RYGB surgery may modulate neurological activity through a miRNA-mediated gut-brain axis. Nine out of these 14 RYGB-altered miRNAs are associated with Parkinson's disease.

### Correlation between miRNAome and the metabolic phenotype post RYGB surgery

**Figure 2:**
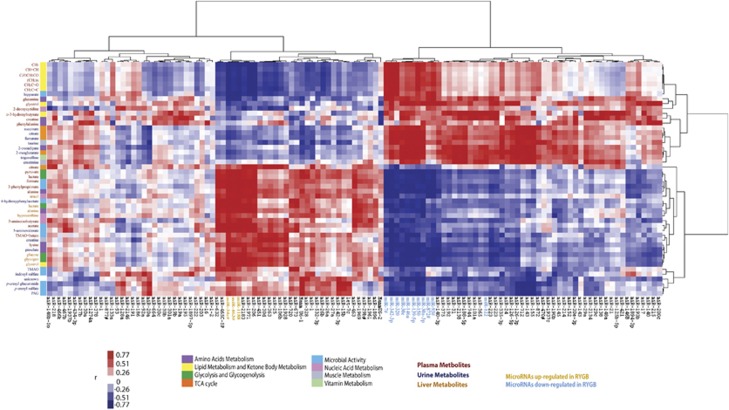
Correlation between miRNome and metabolic profiles. The heat map is generated from the Pearson correlation coefficient values between circulating microRNA expression and metabolite levels using a two-way clustering method. The horizontal axis shows all detectable circulating microRNAs, whereas the vertical axis exhibits altered plasma, urinary and hepatic metabolites following RYGB. The color bar next to the metabolites indicates metabolic functions (amino acid metabolism, lipid metabolism and ketone body metabolism, glycolysis and glycogenolysis, TCA cycles, microbial activity, nucleic acid metabolism, muscle metabolism and vitamin metabolism) of the metabolites. MicroRNAs, which are significantly altered by RYGB surgery, are shown in yellow (upregulated) and blue (downregulated), respectively. SHAM *n*=4, RYGB *n*=4. Keys: PAG, phenylacetylglycine; TMAO, trimethyalmine-*N*-oxide; TCA, tricarboxylic acid.

Following the analysis of predicted miRNA-targeted pathways, we investigated the complex correlations between the circulating miRNAome and the plasma, urinary and hepatic metabolomes. We found that a number of distinctive sets of miRNAs from observed global circulating miRNAs were strongly correlated with plasma lipids, TCA cycle intermediates, host-gut microbial co-metabolites, liver and plasma energy metabolites (Figure 2) and gut hormones (Supplementary Figure S4). Downregulated plasma miR-342-3p and upregulated miR-34c* in RYGB-operated rats demonstrate a broad correlation with the liver, plasma and urinary metabolite profiles, indicating their extensive involvement in metabolic processes. Furthermore, miR-206, miR-1188, miR-1971 and miR-34c* are inversely correlated with plasma lipid fractions, whereas miR-320 and miR-342-3p and to a lesser extent miR-7*, exhibit a positive correlation. TCA cycle intermediates including citrate, succinate, 2-oxoglutarate and fumarate are positively correlated with miR-143, miR-126-3p, miR-146a, miR-150 and miR-155. Interestingly, these same miRNAs are inversely correlated with urinary host-microbial co-metabolites such as *p*-cresyl glucuronide, *p*-cresyl sulfate and phenylacetylglycine. In addition to miR-342-3p and miR-34c*, miR-872*, miR-463*, miR-30e, miR-2183, miR-1971, miR-150, miR-146a, miR-1188 and miR-93* all correlate with liver energy metabolism processes, such as glycolysis and glycogenesis involving glucose, glycogen and lactate.

### Altered miR-122 expression levels in plasma and liver tissues are responsible for metabolic changes post RYGB surgery

**Figure 3:**
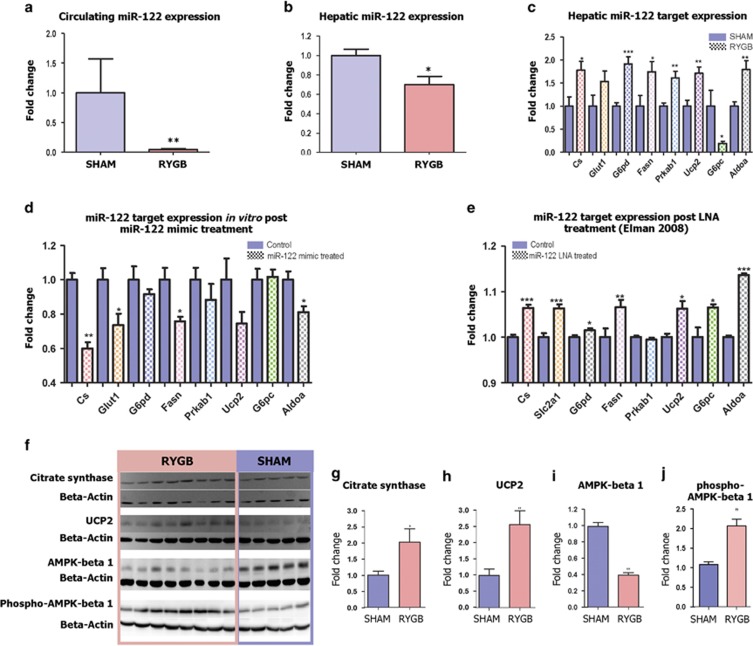
Expression levels of miR-122 and its targets *in vitro* and *in vivo*. The Roux-en-Y gastric bypass results in the altered miR-122 expression levels in plasma (**a**) and the liver (**b**) measured by quantitative PCR. (**c**) The expression levels of miR-122 mRNA targets, including citrate synthase (Cs), glucose transporter protein type 1 (Glut1), glucose-6-phosphate dehydrogenase (G6pd), fatty-acid synthase (Fasn), AMP-activated kinase beta 1 (Prkab1), uncoupling protein 2 (Ucp2), glucose-6-phosphatase (G6pc) and aldolase A (Aldoa), are measured by quantitative PCR in the liver of *RYGB- (n*=8) and *SHAM- (n*=5) operated animals. (**d**) These targets were evaluated in B13H cells transfected with miR-122 mimic (treatment, *n*=4) or scrambled miRNA (control, *n*=4) and measured by quantitative PCR. (**e**) A previous *in vivo* study by Elamn *et al* (Data were extracted from Array Express Experiment No. E-MEXP-1406, *n*=5 each group). (**f**) Immunoblot of the hepatic protein expression levels of citrate synthase, UCP2, AMPK-beta 1 and phosporylated AMPK-beta 1 in the RYGB- and sham-operated animals. (**g**–**j**) show quantified protein expressions of citrate synthase, UCP2, AMPK-beta 1 and phospho-AMPK-beta 1, respectively. All data represent mean±s.e.m. (RYGB, *n*=8; SHAM, *n*=5), unless otherwise specified in each figure. *P<0.05; **P<0.01; ***P<0.001.

RYGB induced the strongest effects on miR-122, as downregulation of miR-122 was observed in both bariatric patients^13^ and our rodent model for RYGB, we subsequently focused on investigating miR-122-mediated metabolic pathways. We first confirmed miR-122 expression levels in both plasma and liver tissue using Taqman real-time quantitative PCR (Figure 3a). The Pearson correlation value of the results derived from Taqman low-density array cards and quantitative PCR is 0.9983 (*P*<0.0001). Plasma miR-122 showed a 99% reduction in levels in RYGB-operated rats compared with SHAM controls, whereas hepatic miR-122 only showed 30% reduction of expression. We next examined a group of miR-122 targets, which are involved in key liver metabolic processes, such as the TCA cycle (*Cs*, citrate synthase), glucose transport (*Glut1*, glucose transporter protein type 1), pentose phosphate pathway (*G6pd*, glucose-6-phosphate dehydrogenase), fatty-acid synthesis (*Fasn*, fatty-acid synthase), energy sensing (*Prkab1*, adenosine monophosphate (AMP)-activated kinase beta 1), mitochondrial oxidation (*Ucp2*, uncoupling protein 2), gluconeogenesis (*G6pc*, glucose-6-phosphatase) and glycolysis (*Aldoa,* aldolase A). All mRNA targets are bona fide validated except for the putative targets UCP2 and AMPKβ1. These miR-122 targets in the liver all exhibited >1.5 fold increase in expression ranging from 1.53 to 1.91, except for *Glut1* and *G6pc*. Although the alteration of *Glut1* was not significant, it showed a clearly increased trend in the expression (*P*=0.1061). *G6pc* expression, a validated miR-122 target, is surprisingly reduced >80% in the RYGB group (Figure 3c). *G6pc* expression was lowered rather than elevated in the hepatic tissue, suggesting perhaps regulatory mechanisms of *G6pc* other than miR-122 predominate. We then investigated whether miR-122 can directly modulate these mRNA targets by manipulating their expression in the hepatocyte-like B13H cell line (derived from a pancreatic AR42J-B13 cell line transdifferentiated with dexamethasone as primary hepatocytes are phenotypically unstable over the required time frame for the experiment). Transdifferentiation of this pancreatic cell to the B13H cell derivative, results phenotypically in a liver-like cell expressing liver-specific and enriched functional enzymes.^14^ In fact, miR-122, believed to be a hepatocyte-specific miRNA, was upregulated more than 400-fold during this trans-differentiation process (Supplementary Figure S5), consistent with the hepatic phenotype. In B13H cells transfected with miR-122 mimic, expression of *Cs*, *Glut1*, *Fasn, Aldoa, G6pd, Prkab1* and *Ucp2* were all reduced (although the last three not significantly) in comparison with controls transfected with scrambled miRNA mimic (Figure 3d). Consistent with the aforementioned *in vivo* findings, the *G6pc* expression level was not affected by miR-122 in the B13H cell line. We concluded that miR-122 directly modulated *Cs*, *Glut1*, *Fasn, Aldoa* and to a lesser extent, *G6pd*, *Ucp2* and *Prkab1*.

We confirmed these transcriptional effects on protein expression in the liver tissues of RYGB- and SHAM-operated rats (Figure 3f). CS, catalyzing the formation of citrate from oxaloacetate and acetyl coenzyme A, is a mitochondrial marker and has a key role in regulating energy generation in TCA cycle and mitochondrial respiration. We found that RYGB rats exhibited a twofold increase in CS protein expression (Figure 3g). UCP2 protein level showed a 2.6-fold upregulation in the RYGB animals (Figure 3h), consistent with the altered mRNA expression level (1.7-fold upregulation). AMPK acts as an energy switch; in an energy-deficient status (high AMP:ATP; adenosine triphosphate ratio), AMPK can switch off ATP-consuming biosynthesis pathways, such as *Fasn* in adipocytes, cholesterol synthesis and gluconeogenesis in the liver, and switch on ATP-producing catabolic pathways, for example, fatty-acid oxidation and glycolysis in multiple tissues.^15^ AMPKβ1 units require activation via phosphorylation.^16^ Although the non-phosphorylated-AMPKβ1 was downregulated by 2.5 fold, the active phosphorylated-AMPKβ1 protein was significantly upregulated by twofold (Figures 3i and j).

## Discussion

Bariatric surgery has been shown to markedly alter circulating miRNAs in patients, in particular, downregulation of miR-122 by 94.7%.^13^ However, the metabolic functions and consequences of these dysregulated miRNAs have not been clarified or validated to date. We therefore investigated the correlation of the miRNAome and metabolites in a bariatric surgical model to elucidate energetic, microbial and neuronal signaling mechanisms of disease resolution. The mechanisms of RYGB surgery are complex; from our study and the work of others; it is apparent that circulating miRNAs could potentially work as a class of hormone, so-called miRormone,^17^ to mediate energy metabolism resulting in weight loss and potentially reset the brain-food rewarding system. Decreased plasma levels of miR-342-3p, miR-320, miR-139-5p and miR-146a observed in our study suggest RYGB surgery impact on multiple neurodegenerative disease-related pathways. Patients with Parkinson's disease commonly experience unintended weight loss^18^ and factors including reduced food intake owing to dysphagia, increased resting energy expenditure and potential peptide hormones, such as ghrelin, could account for the underlying mechanisms.^19^ There is increased evidence suggesting a beneficial effect of bariatric operations on neurodegenerative disease based on surgical activity on caloric restriction, glycaemic control and gut hormonal modulation.^20^ The common miRNA-targeted pathways between bariatric surgery and Parkinson's disease suggest that surgery-induced weight loss may share certain mechanistic pathways with unintended weight loss in Parkinson's disease patients.

**Figure 4:**
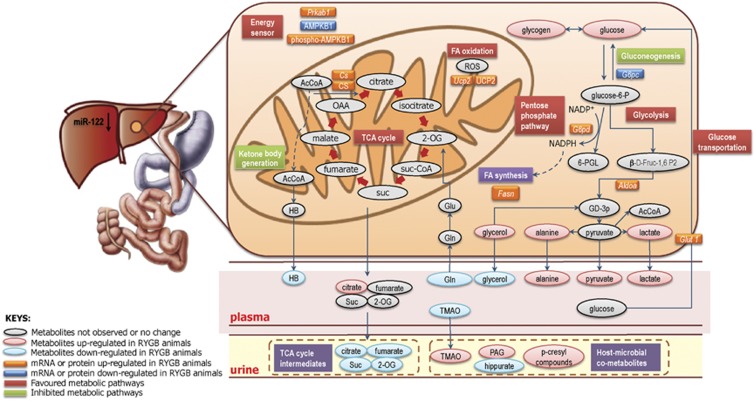
Modulation of metabolic activity by the downregulation of miR-122 following Roux-en-Y gastric bypass surgery. RYGB-induced metabolic changes in liver, plasma and urine (red and blue oval), metabolic enzymes (red and blue 3D boxes), and metabolic pathways (red and green flat boxes) are summarized. Keys: 2-OG, 2-oxoglutarate; 6-PGL, 6-phospho-D-glucono-1,5-lactone; AcCoA, Acetyl coenzyme A; Alodoa, aldolase A; AMP-activated kinase beta 1 (Prkab1, AMPK beta 1), β-D-Fruc-1,6 P2; *Cs*, citrate synthase; FA, fatty acids; *Fasn*, fatty-acid synthase; *G6pc*, glucose-6-phosphatase; *G6pd*, glucose-6-phosphate dehydrogenase; GD-3p, glyceraldehydes-3-phosphate; Gln, glutamine; Glu, glutamate; glucose-6-p, glucose-6-phosphatel; *Glut1*, glucose transporter protein type 1; HB, D-3-hydroxybutyrate; NADPH/NADP+, nicotinamide adenine dinucleotide phosphate; OAA, oxaloacetate; PAG, phenylacetylglycine; ROS, reactive oxygen species; suc, succinate; suc-CoA, succinyl coenzyme A; TCA cycle, tricarboxylic acid cycle; TMAO, trimethylamine-*N*-oxide; Ucp2, uncoupling protein 2.

MiR-122 is a liver-produced miRNA and its less marked reduced expression in the liver compared with plasma suggests a restrained miR-122 release into the blood stream after RYGB surgery. By antagonizing miR-122 with LNA (locked-nucleic-acid-modified oligonucleotide)-anti-miR-122 in diet-induced obese mice for 6 weeks, a 30% decrease in total cholesterol levels was achieved without lipid accumulation in the liver and tested miR-122 mRNA targets were similar to our findings except for *G6pc*, which was upregulated.^21^ Esau *et al.*^22^ demonstrated that injecting miR-122 antisense oligonucleotide into mice for 5 weeks results in reduced plasma cholesterol levels, increased hepatic fatty-acid oxidation, decreased hepatic fatty-acid and cholesterol synthesis rates; effects that are also seen in primates.^23^ Following RYGB surgery, we observed increased glucose transportation, accelerated glycolysis and inhibited gluconeogenesis in the RYGB rat model. *Glut1*, a glucose transporter, was upregulated consistent with the decreased miR-122 expression in the liver and increased levels of hepatic glucose and glycogen were observed, indicating that RYGB promotes glucose transportation and glycogen synthesis. Our metabolic and miR-122 target expression data show that RYGB surgery suppresses gluconeogenesis and stimulates glycolysis, as evidenced by the downregulation of *G6pc* and upregulation of *Aldoa*, together with elevated concentrations of glycolysis end products including pyruvate, alanine and lactate in the liver and plasma (Figure 4). Mencarelli *et al.*^24^ have recently investigated the effects of ileal interposition, where the distal ileum is relocated into the proximal jejunum, which mimics a partial procedure of RYGB surgery. Consistent with our results, the hepatic expression of *G6pc* was also suppressed in this ileal interposition model. Furthermore, *G6pd* was found to be upregulated and this is an indicator for oxidative activity of the pentose phosphate pathway. It is likely that *G6pd* is stimulated by the unbalanced ratio of NADPH/NADP+ (normally 100:1 in the hepatic cytosol) to favor NADPH production. This unbalanced ratio could be because of the utilization of NADPH in the liver by reductive biosynthesis, such as lipid biosynthesis. G6PD expression and enzyme activity have been reported to be upregulated in the Roux limb of RYGB-operated rats, as well as *Glut1*,^25^ and the authors concluded that the reprogramming of intestinal glucose, including enhanced basolateral glucose uptake, augmented glycolysis and stimulated pentose phosphate pathway, could contribute to the glycemic control after RYGB.

Decreased miR-122 levels induced the upregulation of *Cs* (CS) and *Ucp2* (UCP2). CS is a well-studied rate-limiting enzyme in the TCA cycle. Although the metabolic function of UCP2 is still controversial and varies between various types of tissues, it is believed to have a pivotal role in obesity, diabetes, metabolic syndrome and several neurodegenerative diseases^26^^27^ Hepatic UCP2, which uncouples oxidative phosphorylation with ATP production, is highly associated with oxidative phosphorylation and fatty-acid oxidation. UCP2 is responsible for proton transportation from the intermembrane space to the mitochondria matrix and uncouples proton flux with ATP synthesis. The upregulation of UCP2 and CS in the liver indicates that the RYGB-operated animals exhibit higher cellular metabolic activity such as favored fatty-acid oxidation and increased TCA cycle metabolism, which is further supported by the decreased levels of urinary TCA cycle intermediates detected by metabonomic analysis. Furthermore, UCP2 is a downstream effector of the energy sensor AMPK coded by the Prkab1 gene. In the present study, we also observed increased *Prkab1* expression followed by upregulated UCP2. After RYGB, fat stored in adipose tissue is thought to be mobilized and metabolized in the liver,^28^ generating increased levels of free fatty acids, which could result in upregulated UCP2. It has been shown that increased UCP2 could not only promote the energy expenditure toward lipid utilization^29^ and compensate reduction of reactive oxygen species,^30^ but also reduce oxidative stress^31^ and protect mitochondria during fatty-acid β-oxidation.^32^ This is consistent with decreased reactive oxygen species reported in patients after bariatric surgery.^33, 34^ Hence, UCP2 and oxidative decoupling may contribute to postoperative weight loss. We observed that fatty-acid synthase (*Fasn*) also increased following RYGB surgery. The underlying reason for the increase in *Fasn* is unclear, but one possibility is that RYGB extensively affects lipid metabolism and leads to a restoration of adipose redox balance. Therefore, further studies should address lipid metabolism during the re-adjustment period after bariatric surgery.

In conclusion, we have demonstrated that RYGB surgery significantly modified metabolite and miRNA profiles of SD rats, resulting in substantial weight loss. We have demonstrated that miR-122 contributed to the control of energy metabolism with increased glucose transportation, glycolysis, TCA cycle, pentose phosphate shunt and fatty-acid oxidation and decreased gluconeogenesis and ketone body generation (Figure 4), suggesting an overall increased energy expenditure status. By correlating the circulating miRNAome and metabolome data, we were able to generate for the first time a comprehensive landscape of the crosstalk between miRNAs and metabolic pathways that point to a systemic regulation of processes including multiple axes of energy metabolism and suggested impact on the gut-brain axis. Follow-up studies will allow a detailed understanding of miRNAs responsible for regulating specific metabolic pathways, and conversely identifying metabolites capable of regulating the expression and activity of specific miRNAs. Our study highlights the value of transomic modeling and opens up a new window to further understand fundamental mechanisms associated with metabolic surgery and potential therapeutic targets for obesity and diabetes treatment.

## Figures and Tables

**Table 1:** Plasma, urinary and hepatic aqueous metabolites found to be altered between RYGB- and SHAM-operated rats based on OPLS-DA models

*Plasma metabolite*	*An OPLS-DA model of plasma CPMG spectral data (Q*^*2*^*Y=0.83; R*^*2*^*X=33.5%)*			
	*Change in RYGB*	*Peak (ppm)*	P*-value*	q*-value*
CH_3_	↓	0.87	0.002	0.025
(CH_2_)*n*	↓	1.266	0.005	0.03
CH_2_CH_2_CO	↓	1.562	0.006	0.03
CH_2_C=C	↓	2.036	0.002	0.02
CH_2_C=O	↓	2.227	0.004	0.03
CH=CH	↓	5.3	0.007	0.03
Pyruvate	↑	2.357	0.006	0.03
Lactate	↑	4.107	0.004	0.03
D-3-hydroxybutyrate	↓	1.178	0.09	0.14
Glutamine	↓	2.426	0.003	0.03
Citrate	↑	2.664	0.02	0.06
Creatine	↓	3.023	0.004	0.03
TMAO+betain	↓	3.252	0.004	0.03
Glycerol	↓	3.566	<0.001	0.01
2-deoxycytidine	↓	6.04	<0.001	0.02
Formate	↑	8.443	<0.001	0.003
Phenylalanine	↓	7.407	0.004	0.03
Acetate	↑	1.904	0.03	0.06
2-phenylpropionate*	↑	1.425	<0.001	0.005
Alanine	↑	1.471	<0.001	0.005
Lysine	↑	1.892	<0.001	0.006
3-aminoisobutyrate*	↑	1.2	0.004	0.03

*Urinary metabolite*	*An OPLS-DA model of urinary spectral data (Q*^*2*^*Y=0.93; R*^*2*^*X=36.8%)*			
	*Change in RYGB*	*Peak (ppm)*	P*-value*	q*-value*
Succinate	↓	2.413	<0.001	<0.001
2-oxoglutarate	↓	2.448	<0.001	<0.001
Citrate	↓	2.548	<0.001	<0.001
Fumarate	↓	5.528	<0.001	<0.001
2-oxoadipate	↑	1.824	<0.001	0.002
5-aminovalerate	↑	1.645	0.05	0.06
Pimelate	↑	1.31	0.004	0.01
TMAO	↑	3.273	0.002	0.007
Taurine	↓	3.433	0.002	0.006
Creatine	↑	3.933	0.1	0.09
Creatinine	↓	4.054	<0.001	0.003
4-hydroxyphenylacetate	↑	3.454	0.001	0.004
Unknown	↑	3.523	0.002	0.006
*p*-cresyl glucuronide	↑	2.301	0.002	0.005
*p*-cresyl sulfate	↑	2.346	0.002	0.007
PAG	↑	3.757	<0.001	<0.001
Hippurate	↓	3.974	0.009	0.02
Trigonelline	↓	4.436	<0.001	<0.001
Indoxyl sulfate	↑	7.714	<0.001	0.002

*Liver metabolite*	*An OPLS-DA model of liver aqueous extract (Q*^*2*^*Y=0.81; R*^*2*^*X=45.6%)*			
	*Change in RYGB*	*Peak (ppm)*	P*-value*	q*-value*
Lactate	↑	1.339	0.001	0.005
Alanine	↑	1.479	0.01	0.02
Glucose	↑	5.238	<0.001	0.002
Glycogen	↑	5.415	<0.001	0.002
Glycerol	↑	3.78	<0.001	0.001
Uracil	↑	5.803	0.03	0.05
Hypoxanthine	↑	8.2	0.006	0.02

Abbreviations: CPMG, Carr-Purcell-Meiboom-Gill; OPLS-DA, orthogonal-partial least squares-discriminant analysis; PAG, phenylacetylglycine; RYGB, Roux-en-Y gastric bypass; TMAO, trimethylamine-*N*-oxide.

R^2^X represents the variation in ^1^H NMR spectral data explained by the OPLS-DA model. Q^2^Y indicates the level of significance of the metabolic differences between two classes. The *P-* and *q-*values in the table represent the significance of the metabolite changes and false discovery rate-adjusted *P*-values, respectively. The arrow up or arrow down represents the increased or decreased trend of metabolites in RYGB-operated rats compared with SHAM controls. *Tentative metabolite assignment.
