# Supplementary material for: Metabolic phenotype-microRNA data fusion analysis of the systemic consequences of Roux-en-Y gastric bypass surgery
Source: Int J Obes (Lond). 2015 Apr 28;39(7):1126–34. doi: 10.1038/ijo.2015.33 (PMC4766927; doi:10.1038/ijo.2015.33)
Supplement: Supplementary Information [file ijo201533x1.docx]

**Supplementary Information**

**Materials and Methods**

**Liver sample homogenization and metabolite extraction.** The liver tissue samples (≈100 mg) were put into a 7 ml vial containing 1.2 ml of water (HPLC grade) and homogenized using a tissue homogenizer at 135,000 Hz. A total of 0.6 ml of the homogenized mixture was transferred into a 1.5 ml Eppendorf tube for further RNA extraction. The remaining 0.6 ml was transferred into a glass test tube for metabolite extraction using pre-chilled water, methanol and chloroform. Approximately 3.5 ml of water was added into the test tube, vortexed for 30 sec, and 1.25 ml of chloroform and 0.75 ml of methanol was added followed by another 30-sec vortex. The samples were left on ice for 10 min, allowing the metabolites to dissolve thoroughly and then vortexed for 30 sec prior to the centrifugation at 4 °C for 10 min at 1,585 x *g*. The aqueous and organic layers were transferred into vials, separately. The extraction procedure was repeated twice on the remaining pellet and the aqueous and chloroform phases from the same sample were combined with previous phases. The aqueous phase was dried using a speed vacuum centrifuge and the organic phase was left in the fume hood to dry overnight.

**Sample preparation for NMR spectroscopic analyses**. Plasma samples collected using sodium heparin and urine were thoroughly defrosted and vortexed for 15 sec. A total of 30 μl of urine was mixed with 25 μl of 0.2 M sodium phosphate buffer in D_2_O (0.01% of sodium 3-(trimethylsilyl) propionate-2,2,3,3-*d*_4_ [TSP], pH=7.4), and 50 μl of the mixture was transferred into an NMR tube with an outer diameter of 7 mm for further spectroscopic analysis. A total of 400 μl of plasma was mixed with 250 μl of saline containing 20% deuterium oxide (D_2_O) for the magnetic field lock. The resulting mixture was centrifuged at 10,000 x *g* for 10 min and 600 μl of supernatant was transferred into a NMR tube with an outer diameter of 5 mm pending ^1^H NMR spectral acquisition. The dry extracts of liver aqueous phase were resuspended in 600 μl of the aforementioned sodium phosphate buffer, centrifuged for 10 min at 10,000 x *g* and 600 μl of supernatant was transferred into a NMR tube.

**Two-dimensional ^1^H Nuclear magnetic resonance spectroscopy of urine, plasma and liver extracts.**

A series of 2-D NMR spectra including ^1^H-^1^H correlation spectroscopy (COSY) and ^1^H-^1^H total correlation spectroscopy (TOCSY) were acquired on the selected urine and faecal extract samples for the purpose of metabolite annotations. The standard parameters for these spectral acquisitions were previously reported (1).

**^1^H NMR spectral data processing and multivariate statistical analysis.** ^1^H NMR spectra of urine, plasma and liver aqueous extracts were manually phased, referenced (to TSP at δ 0.0 in urinary and liver aqueous extract spectra and to anomeric α-glucose proton at δ 5.223 in plasma spectra) and baselines were corrected in TopSpin 3.0 (Bruker, Germany). The resulting NMR spectral data (δ0-10) were imported to MATLAB software and binned into 20 K data points with the resolution of 0.0005 ppm using a script developed in house (Dr. O. Cloarec). The water peak region (δ-4.62-5.05) was removed in order to minimise the effect of the artificially disordered baseline. Probabilistic normalisation was performed on the remaining spectral data in order to take into account differences in dilution factor and tissue weight. Principal component analysis (PCA) and OPLS-DA (orthogonal partial least squares-discriminant analysis) (2) were carried out on the resulting NMR spectral datasets using MATLAB (2012a). Metabolites identification was also aid by Statistical TOtal Correlation SpectroscopY (STOCSY) (3).

**Single Taqman microRNA assay.** In order to validate individual miRNAs, Taqman microRNA assay was used. RNA solution (5µL) from 100µL elute was used as input into each reverse transcription reaction. Under the conditions of the extraction, 5 µL of final RNA solution was derived from 5 µL of plasma. The RNA was reverse transcribed by Taqman microRNA reverse transcription (RT) kit and Taqman microRNA stem loop primers (Applied biosystems). RT product (1.6µL) was then combined with 10 µL TaqMan® Fast Universal PCR Master Mix II (2×), no UNG, 1 µL TaqMan® Small RNA Assay (20×) and 7.67 µL water to generate final 20 µL volume. Each qPCR was performed in triplicate using an Applied Biosystem 7500HT fast system.

**mRNA target prediction.** A target pathway was derived using Panther software, which calculated p values by a binomial statistic method (4). Prediction of the mRNA targets of each significantly changed miRNA was made using nine commonly used databases, namely, miRWALK, DIANAlab, miRanda, miRDB, PICTAR4, PICTAR5, PITA, RNA22 and Targetscan, and only targets predicted by more than two databases were included. The predicted pathways, affected by RYGB surgery, are ranked according to the number of miRNAs involved in each pathway.

**Protein extraction from the liver and Immunoblot.** An extract of whole liver was prepared in RIPA buffer (SIGMA-Aldrich). Approximately 50mg liver tissue from RYGB and SHAM operated rats were homogenized with 500 µL PBS contained 1% protease inhibitor cocktail (SIGMA-Aldrich). Samples were then centrifuged at 13500 g for 5 minutes. Pellets were re-suspended in 400 µL RIPA buffer and sonicated for 30 minutes at 4ºC. Protein was collected by centrifugation and dissolved in water. Twenty μg of protein sample were separated by 10% SDS-PAGE gel. The protein was electro-transferred from the gel onto wet nitrocellulose membrane. Nonspecific binding sites were blocked for 1 hour with blocking buffer (PBS-tween solution with 5% milk powder) at room temperature. Primary antibodies were incubated for 1 hour at room temperature and secondary antibodies were incubated at 4^o^C overnight. The following primary antibodies were used: citrate synthase (Abcam, ab129095), uncoupling protein 2 (Abcam, ab67241), AMP-activated protein kinase (Abcam, ab32112), anti-β actin antibody (Sigma-Aldrich). Secondary anti-mouse and anti-rabbit polyclonal antibodies were purchased from Abcam. Membranes were washed and visualized by chemiluminescent regent (Merck Millipore) with BioRad imaging system.

**MiRDIAN miR-122 mimic transfection.** MiRDIAN miR-122 mimic (C-320349-05-0005) and scrambled microRNA negative mimic control (CN-001000-01-05) were purchased from Thermo Scientific. Pancreatic derived B13 cells were plated 10^5^ in 24 well plates and dexamethasone was used to transdifferentiate the cells into hepatocyte-like cells over 2 weeks as previously described (5). Cells were transfected using Lipofectamine 2000 (Invitrogen). Transfection complex was prepared according to the manufacture’s instructions. Cells were treated in 24 well plates containing 0.5μL miR-122 mimic / negative control (20 μM), 3 μl lipofectamine 2000 and 500 μL of Opti-MEM reduced serum media. Cells were harvested 96 hours later with PBS wash before harvesting.

**Statistical correlation analysis among gut hormones, miRNAome and metabolome.** Pearson correlation between metabolome and miRNAome was calculated in MATLAB (2012a). Two-way clustering analysis was performed on the correlation values and visualised in a heat map using Cluster 3.0 and Java Tree View software. Three-dimensional correlation between gut hormones (GLP-1, PYY), metabolites and miRNAs were performed using Pearson correlation and absolute correlation values >0.65 and p values <0.05, plotted as a heat map.

**References**

1. Beckonert O et al. (2007) Metabolic profiling, metabolomic and metabonomic procedures for NMR spectroscopy of urine, plasma, serum and tissue extracts. Nat Protoc 2:2692–2703.

2. Trygg J and Wold S (2002) Orthogonal projections to latent structures (O-PLS). J. Chemometrics 16(3):119-128.

3. Cloarec O et al. (2005) Statistical total correlation spectroscopy: an exploratory approach for latent biomarker identification from metabolic 1H NMR data sets. Anal Chem 77(5):1282-1389

4. Mi H, Thomas P (2009) PANTHER pathway: an ontology-based pathway database coupled with data analysis tools. Methods Mol Biol 563:123–140.

5. Probert et al (2014) Utility of B-13 progenitor-derived hepatocytes in hepatotoxicity and genotoxicity studies. Toxicol. Sci. 137(2):350-370.

**Table S1.** Summary of all detectable circulating microRNAs.

| Only present in SHAM |  | Only present in RYGB |  | Common | |
| --- | --- | --- | --- | --- | --- |
| mmu-miR-122 |  | hsa-miR-206 |  | hsa-miR-140-3p | mmu-miR-1971 |
| mmu-miR-130a |  | hsa-miR-421 |  | hsa-miR-200c | mmu-miR-19a |
| mmu-miR-197 |  | mmu-miR-1188 |  | hsa-miR-214 | mmu-miR-19b |
| mmu-miR-222 |  | mmu-miR-15b |  | hsa-miR-223 | mmu-miR-20a |
|  |  | mmu-miR-1928 |  | hsa-miR-30a-3p | mmu-miR-2134 |
|  |  | mmu-miR-194 |  | hsa-miR-30e-3p | mmu-miR-2138 |
|  |  | mmu-miR-1961 |  | hsa-miR-93# | mmu-miR-21 |
|  |  | mmu-miR-203 |  | Mamm U6 | mmu-miR-215 |
|  |  | mmu-miR-2146 |  | mmu-let-7c | mmu-miR-223 |
|  |  | mmu-miR-2183 |  | mmu-miR-106a | mmu-miR-24 |
|  |  | mmu-miR-218 |  | mmu-miR-106b | mmu-miR-25 |
|  |  | mmu-miR-30d |  | mmu-miR-125b-5p | mmu-miR-26a |
|  |  | mmu-miR-34b-3p |  | mmu-miR-126-3p | mmu-miR-26b |
|  |  | mmu-miR-34c# |  | mmu-miR-126-5p | mmu-miR-27a |
|  |  | mmu-miR-363 |  | mmu-miR-1274a | mmu-miR-27b |
|  |  | mmu-miR-434-3p |  | mmu-miR-1-2-AS | mmu-miR-29a |
|  |  | mmu-miR-463# |  | mmu-miR-133a | mmu-miR-29b# |
|  |  | mmu-miR-467b |  | mmu-miR-138 | mmu-miR-29c |
|  |  | mmu-miR-532-3p |  | mmu-miR-139-5p | mmu-miR-301a |
|  |  | mmu-miR-685 |  | mmu-miR-140 | mmu-miR-30a |
|  |  | mmu-miR-694 |  | mmu-miR-142-3p | mmu-miR-30b |
|  |  | mmu-miR-712 |  | mmu-miR-1 | mmu-miR-30c |
|  |  | mmu-miR-721 |  | mmu-miR-145 | mmu-miR-30e |
|  |  | mmu-miR-877# |  | mmu-miR-146a | mmu-miR-31 |
|  |  | rno-miR-190b |  | mmu-miR-148a | mmu-miR-320 |
|  |  |  |  | mmu-miR-150 | mmu-miR-328 |
|  |  |  |  | mmu-miR-152 | mmu-miR-335-3p |
|  |  |  |  | mmu-miR-155 | mmu-miR-342-3p |
|  |  |  |  | mmu-miR-16 | mmu-miR-375 |
|  |  |  |  | mmu-miR-17 | mmu-miR-451 |
|  |  |  |  | mmu-miR-186 | mmu-miR-463 |
|  |  |  |  | mmu-miR-188-5p | mmu-miR-465C-5P |
|  |  |  |  | mmu-miR-1894-3p | mmu-miR-466k |
|  |  |  |  | mmu-miR-1896 | mmu-miR-652 |
|  |  |  |  | mmu-miR-1897-5p | mmu-miR-673 |
|  |  |  |  | mmu-miR-1904 | mmu-miR-720 |
|  |  |  |  | mmu-miR-191 | mmu-miR-744 |
|  |  |  |  | mmu-miR-192 | mmu-miR-872 |
|  |  |  |  | mmu-miR-1937b | mmu-miR-92a |
|  |  |  |  | mmu-miR-1937c | rno-miR-1 |
|  |  |  |  | mmu-miR-193b | rno-miR-146B |
|  |  |  |  | mmu-miR-1951 | rno-miR-632 |
|  |  |  |  | mmu-miR-195 | rno-miR-664 |
|  |  |  |  | mmu-miR-1969 | rno-miR-7# |

**Supplementary figure legend**

**Fig. S1.** Body weight and gut hormone levels. **(**S1A) Body weight curve of RYGB- and SHAM-operated animals. (S1B) Circulating gut hormone GLP-1 levels in RYGB- and SHAM-operated animals. (S1C) Circulating gut hormone PYY in RYGB- and SHAM-operated animals. All data represent mean ± SEM (RYGB, n=8; SHAM, n=5). ** p<0.01, *** p<0.0001.

**Fig. S2.** Metabolic changes after Roux-en-Y gastric bypass surgery observed in ^1^H Nuclear Magnetic Resonance (NMR) spectroscopy data from urine (S2A), plasma (S2B) and liver aqueous extracts (S2C). OPLS-DA coefficient loading plot shows the discriminatory metabolites between RYGB- (n=8) and SHAM- (n=5) operated rats. Peaks pointing upwards represent higher levels of the metabolite in RYGB group compared with SHAM group and *vice versa*. The color bar represents correlation coefficient values (r^2^).

**Fig. S3.** The cumulative distribution calculation method of the coefficient of variance (CV) of normalized RQ (fold change). (S2A) The purpose of normalisation is to diminish the within group variance. Generally, delta Ct was individually calculated *via* the formula (raw Ct- normalisation factor). Each individual normalisation defined by specific normalisation factor and the non-normalised method does not subtract any normalisation factors. Subsequently, fold change (RQ) was calculated individually by using the 2^deta Ct divide the mean of 2^deltaCt from the opposite group. Both the experimental RYGB group and the control SHAM group were calculated this way so that RQ CV stands for the all within group variances. These RQ CV scores were then ranked and plotted in S3B. **(**S3B) Cumulative distribution with four normalization methods of miRNA RQ (fold change) CV values.

**Fig. S4.** Three dimensional correlations among MiRNAome, metabolome and gut hormones using Pearson correlation (RYGB, n=4; SHAM, n=4). The Cut off of correlation coefficient values |r|>0.65 and p values <0.05.

**Fig. S5.** MiR-122 expression fold change during B13 to B13H cell transdifferentiation process. Mature miR-122 is detected *via* Taqman quantitative PCR assay. Control (undifferentiated B13) have 6 replicates, all other points have 3 biological replicates. Data represent mean ± SEM.
