# Supplementary figures and images for: Metabolic phenotype-microRNA data fusion analysis of the systemic consequences of Roux-en-Y gastric bypass surgery
Source: Int J Obes (Lond). 2015 Apr 28;39(7):1126–34. doi: 10.1038/ijo.2015.33 (PMC4766927; doi:10.1038/ijo.2015.33)

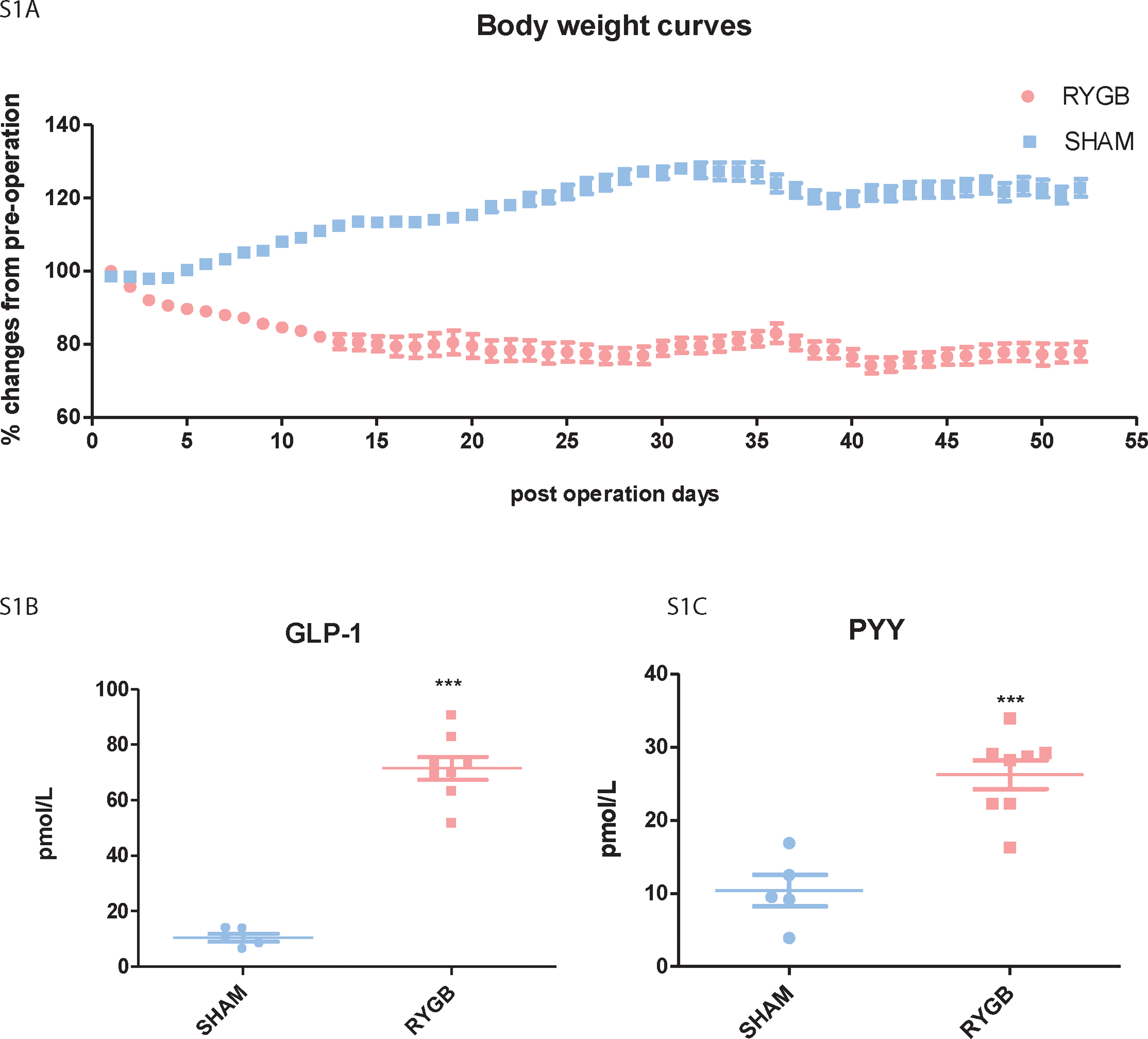

Supplement: Supplementary Figure 1 [file ijo201533x2.tif]

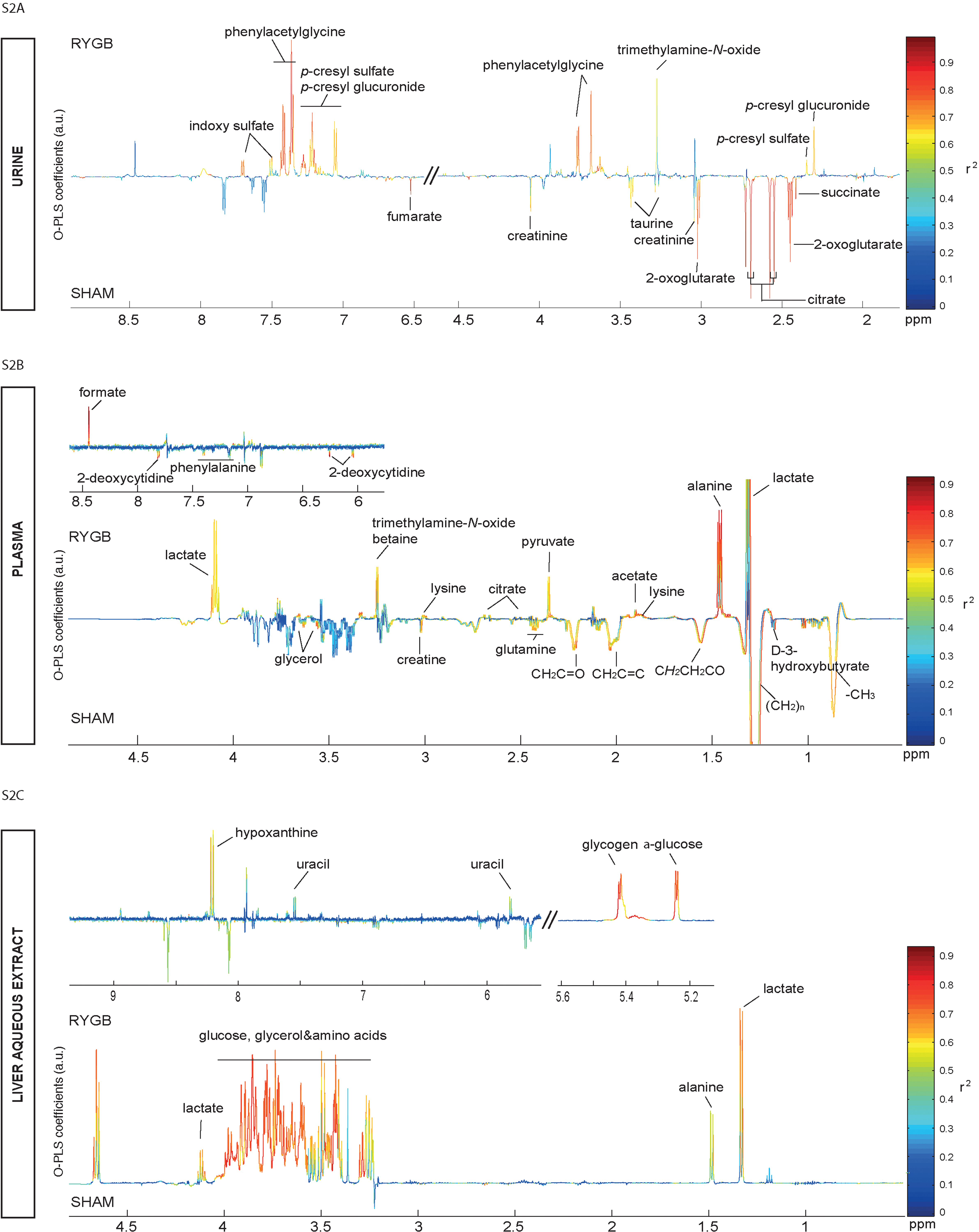

Supplement: Supplementary Figure 2 [file ijo201533x3.tif]

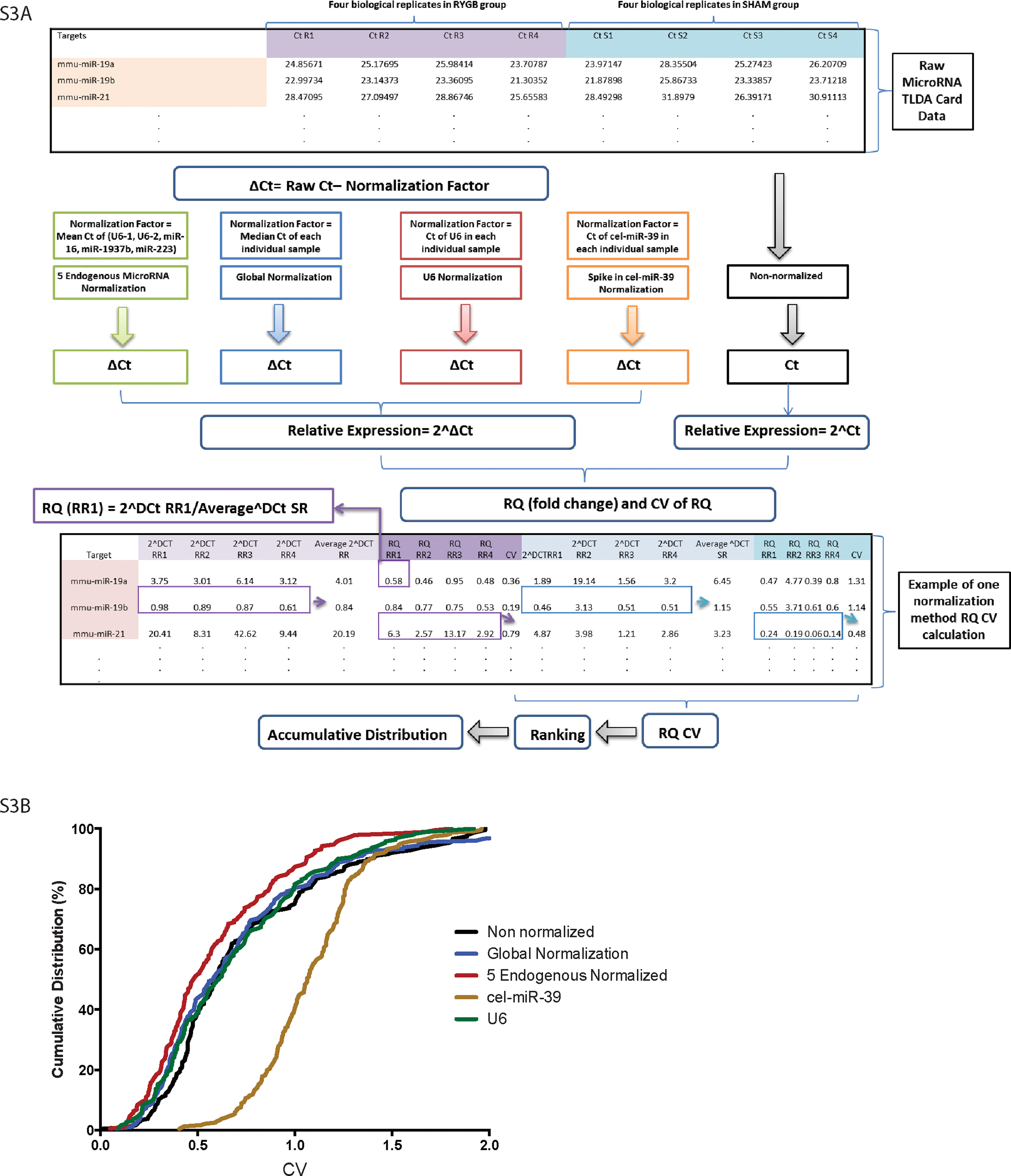

Supplement: Supplementary Figure 3 [file ijo201533x4.tif]

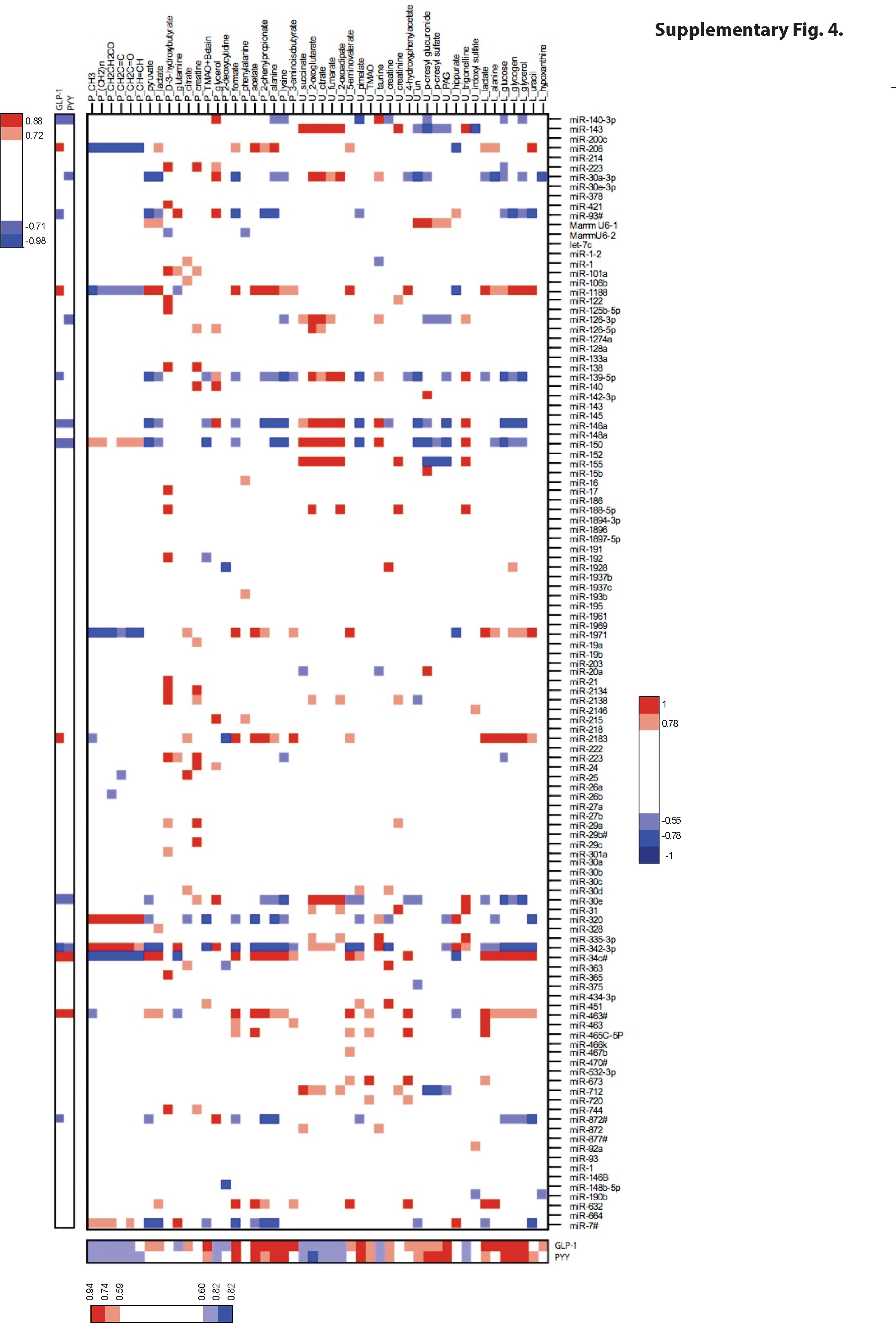

Supplement: Supplementary Figure 4 [file ijo201533x5.tif]

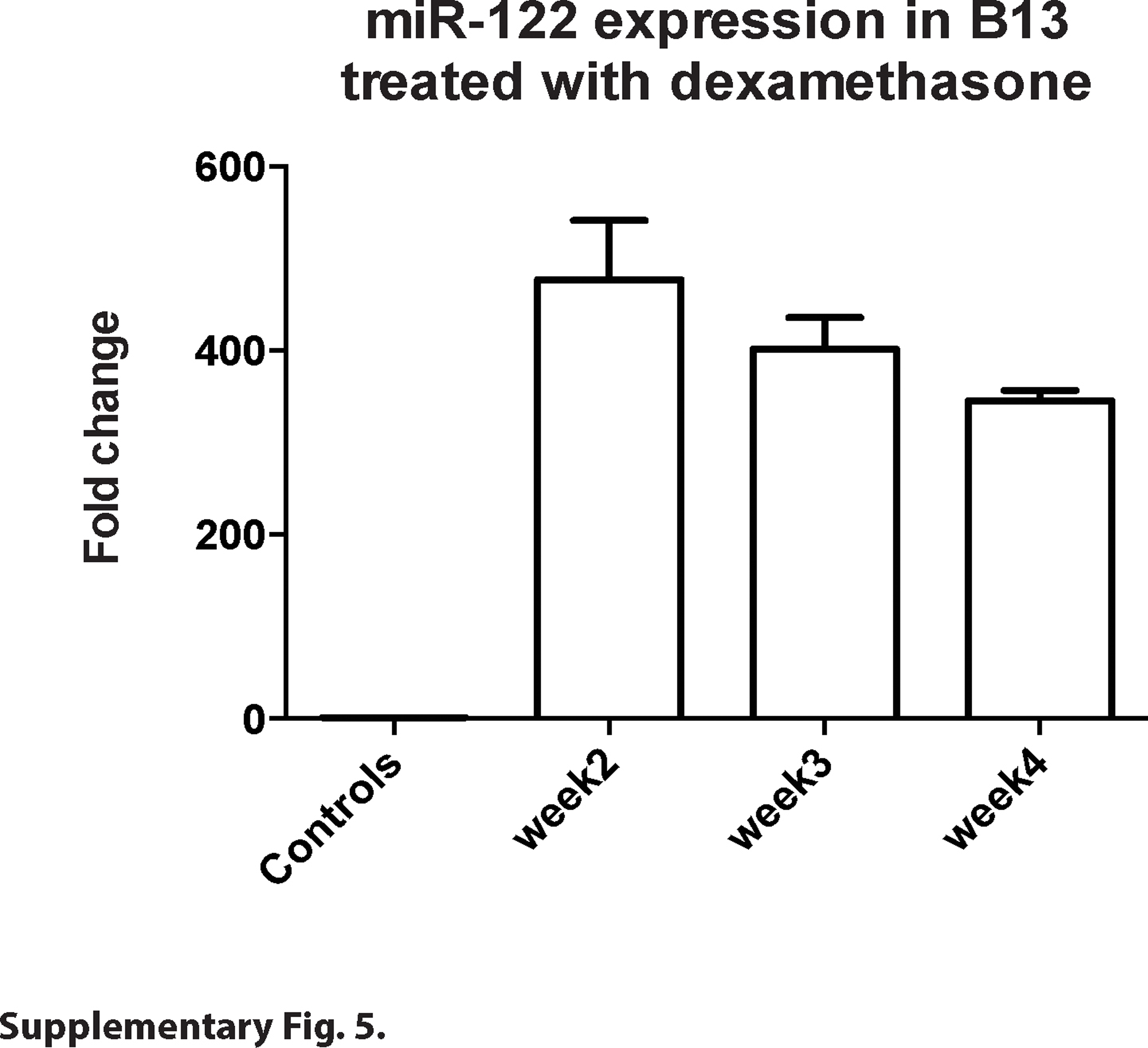

Supplement: Supplementary Figure 5 [file ijo201533x6.tif]
